# Supplementary material for: Antigenic Determinants of SARS-CoV-2-Specific CD4+ T Cell Lines Reveals M Protein-Driven Dysregulation of Interferon Signaling
Source: Front Immunol. 2022 Jun 30;13:883159. doi: 10.3389/fimmu.2022.883159 (PMC9279651; doi:10.3389/fimmu.2022.883159)
Supplement: Supplementary file 1 [file DataSheet_1.pdf]

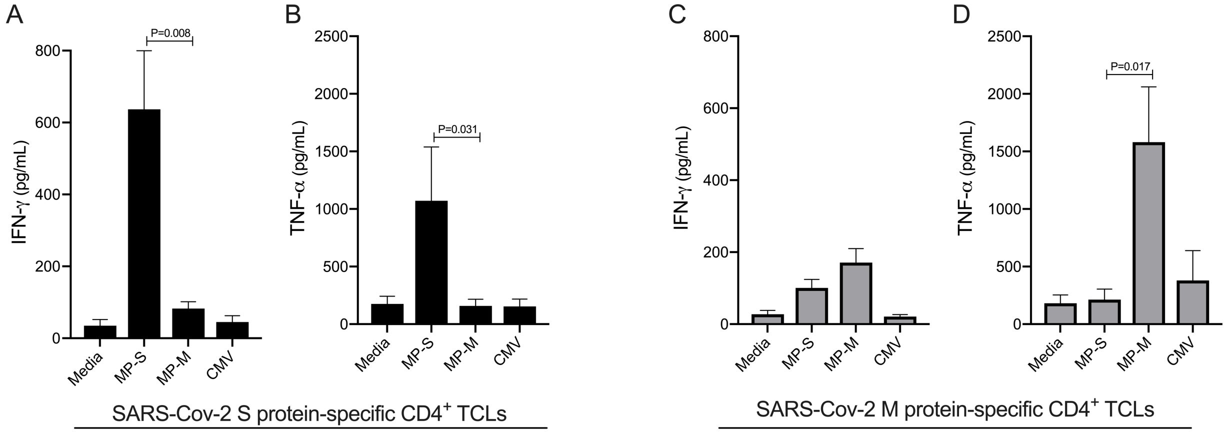

Supplemental figure 1. SARS-CoV-2 S and M proteins-specific CD4$^+$ TCLs reactivity and specificity. 2 x10^5 S-reactive TCLs (A) and 2 x10^5 M-reactive TCLs (B) from the 6 donors were incubated with 2 x10^5 autologous irradiated PBMCs and stimulated separately in the absence (media) or in the presence of MP-S (1µg/mL) and MP-M (1µg/mL) and CMV MP (1µg/mL) for 48 hours at 37°C and 5% CO_2. IFN-γ and TNF-α levels were measured in the culture supernatant by Luminex platform. All differences by Wilcoxon matched-pairs test with $P < 0.05$ are indicated in the graph.

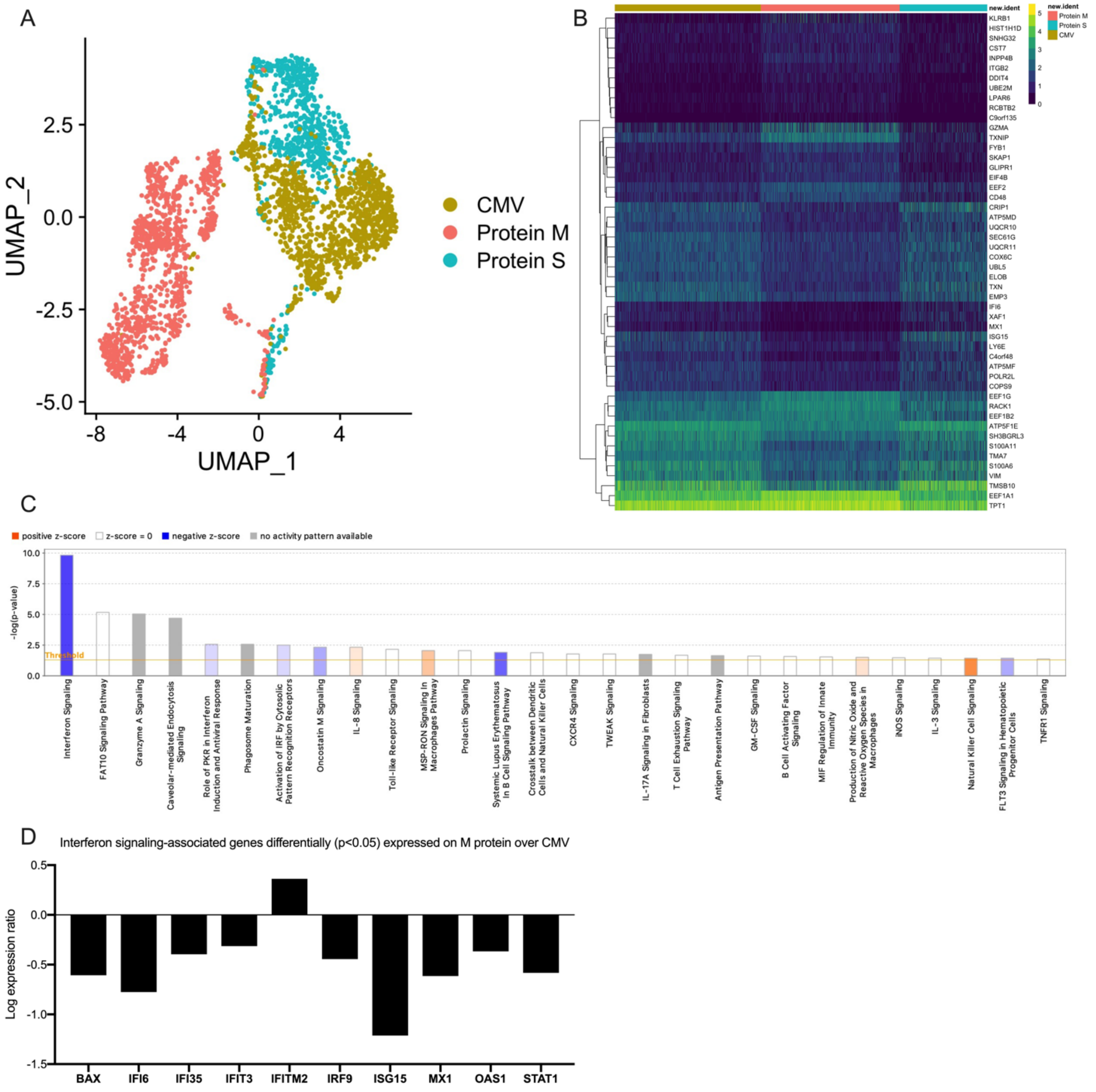

Supplemental figure 2. Single-cell transcriptional profiling of SARS-CoV-2 S, M and CMV-specific $CD4^+$ T cells (A). Heatmap showing expression of the most significantly 50 enriched transcripts in M protein-specific $CD4^+$ T cell lines over S protein- and CMV-specific $CD4^+$ T cell lines (B). Canonical signaling pathways (IPA, QIAGEN) of immunological relevance affected in M protein-specific TCLs over CMV-specific TLCs indicating a marked suppression of interferon signaling pathway (C), with the associated gene expression levels and directions presented individually (D).

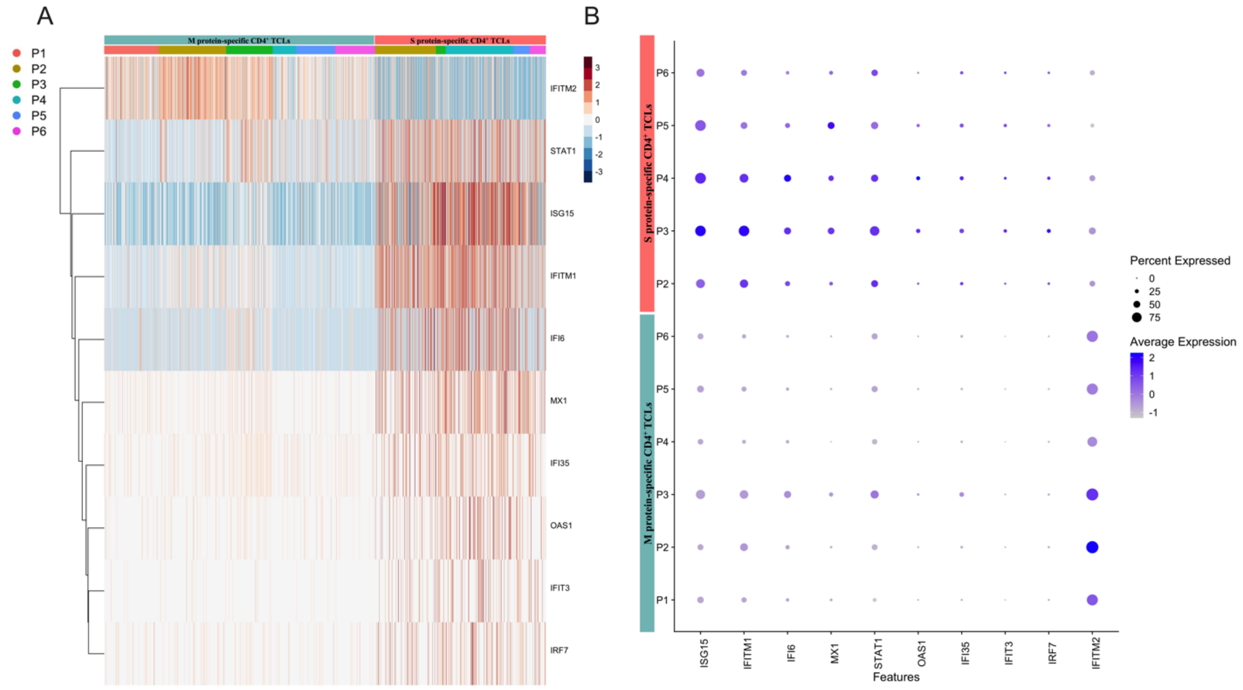

Supplemental figure 3. Heatmap (A) and the feature dot plot graph (B) showing the average expression and the percent expression of the interferon signaling pathway genes in the respective S-protein-specific $CD4^+$ T cell lines and M-protein-specific $CD4^+$ T cell lines by a donor-specific manner (colored from P1-P6).
